# BlockProof: A Framework for Verifying Authenticity and Integrity of Web Content

**DOI:** 10.3390/s22031165

**Published:** 2022-02-03

**Authors:** Meirylene Avelino, Antonio A. de A. Rocha

**Affiliations:** Instituto de Computação, Universidade Federal Fluminense, Niterói 24033-900, Brazil; meirylenerea@id.uff.br

**Keywords:** blockchain, blockchain-based applications, integrity, authenticity, security

## Abstract

In the Literature, we can find several research works to help in the digital crime fight in order to prove integrity and authenticity of a published document, image or video. Among all the crimes, fake news certainly is among the most recurrent ones and needs to be mitigated. There are several Blockchain-based applications in order to make use of the benefits derived from technology, but little is found to verify the authenticity of Web content records as well as the history of all updates that have taken place in each Web content. Such kind of solution has become important nowadays as a way to cover the gap in the combat against fake news, for example. The purpose of this paper is to present BlockProof, a framework for verifying web content authenticity and integrity that offers a solution for content providers to register Web content, regardless of whether the page has dynamic or static content, in addition to enabling the consultation of the history of all records made for a given URL. We understand that such kind of solution may be useful to data producers/providers to provide evidence that they are in compliance with the fight against fake news, for instance.

## 1. Introduction

Internet emerged in the mid-1960s as a means of military purposes communication, expanding and being disseminated throughout the world and, in the 1990s, the Internet began to be used by ordinary users. Over the years, more transformations have been carried out and society has become accustomed to a different way of information consuming, services and content, in a quick manner, any time you need and any place you are. With social media emergence, creation and sharing of diverse subjects were available to anyone. Social networks are one of the strategies used by society to share information and knowledge, through the relationships between actors that integrate them [1].

Through social networks, users can be immersed in an environment, minimally controlled, for exposing their opinions at various subjects, share contents, and in addition they can identify with other people who share similar opinions to his own. Thus, it is possible to feel more inserted in society through these relationships that are built in that media. According to Pew Research Center, 65% of adults use social networks or social networking sites [2].

This represents an increase of almost ten times in the last decade. It is undoubtedly the quickest way to disseminate content and to build an opinion on a given subject. Basically, anyone who has access to the Internet can publish some information, content providers and serious companies that are responsible for distributing information, technologies and services, are increasingly adopting means of ensuring their suitability.

In the past, when there was no Internet and the news about a certain fact was published in print media, for example, if someone published some fake news, it was easy to prove that news was fake and often even a retraction was done because it was easy to prove the information was printed. However, how can we prove today that fake news has been published in digital media? Can this content provider certify to its consumers that all the information that is disclosed through them were in fact published by them?

The Internet becomes a very powerful tool, which allows us to learn and improve our acquired knowledge and information without any speed comparison; on the other hand, it also presents us with a problem: how to guarantee the information authenticity (and with its non-repudiation).

Any published internet act that defames someone’s honor or reputation purpose will receive penalties with the rigor that action requires. The biggest challenge is with the evidence purchase that proves that the crime was undeniably committed. This will have crucial importance in the legal proof, as it involves possible compensation and incontestability evidence presented may be decisive in the verdict of the case.

There is a document that is used when you want to register some digital information as evidence with legal validity, for example: Fake News, screenshots of cell phone messages exchanges. This document is called “The notarial act”. It is a kind of public instrument endowed with public faith, where the report of the fact is captured, and it is transferred to its notebooks or for another document, and the notary may not have any alteration, interpretation or adaptation of the fact, or value judgment.

As much as the notarial act is a widely used resource, there are some points that do not make it exempt from contestation. The description of the fact to be documented is performed unilaterally. The victim describes what happened to the notary public without the presence of a neutral entity to contest the doubtful facts and this can generate possible doubts about the authenticity of this report if the minutes are used in any legal process, for example.

For the content provider’s reputation, which is responsible for servers that host the published news, if this news was needed in the future to be used as crime evidence, it is interesting that there are ways in which the provider can attest to his good reputation wherever content published by it can be certified that it was published by that provider. It is willing, from the perspective of the content provider, to have ways to protect them from content tampering attacks, where a malicious agent can plagiarize a website of the provider, for example, and therefore possible dissemination of fake news, in addition to a means by which providers can attest that a publication was originated by them indisputably.

The sites that store the contents found on the internet are hosted on servers connected to the network where they have a numerical identification known as IP—Internet Protocol. When a user accesses certain content, he accesses it via a URL (Uniform Resource Locator) which “points” to an IP address from the host server of the accessed content. The evidence of crimes involving exposed websites contents may have their authenticity challenged by the defense, and it is necessary to obtain ways to ensure that the collected files is authentic in an irrefutable way. The published web evidence is “volatile” and can be changed and/or deleted with some ease.

Imagine the situation where a user accesses a web page from a service provider. content. At this time, the page content is accessed by the customer browser and the user sees that content being displayed on the screen believing that he is indeed truthful. Suppose the user’s computer has gone through a DNS (Domain Name System) Spoofing attack, which in short is an attack where inserts perform incorrect resolution information for a host that does not have the authority to provide some information. The attacker can put multiple IP addresses that will redirect the legitimate client to a server under the attacker’s control which could be a copy of some website hosted on a legitimate content provider. As the user has no way of knowing that the content accessed is fake, it may sue the legitimate content provider under something he did not disclose. Considering the context presented, can the provider legitimate prove to the user that the information was not generated by him?

These were some of the factors that motivated the development of the Framework proposed in this paper. In an environment where mutual trust between those involved makes it dubious, as there are different ways to spread fake news—tampering with web content linking the origin to a provider that may not actually be responsible for the publication, for example—it is necessary for there to be a third element to authenticate the transactions carried out. As the web environment is a dynamic environment, it is not possible to assign this responsibility only to an agent; for this reason, the Blockchain technology was adopted.

Blockchain has a concept that aims at decentralization as a security measure. The data records are distributed and shared, with the intention of creating a global index for all transactions that occur in each network. It works as a ledger in a public shared and universal way, which creates consensus and trust in direct communication between two parties without the third party’s intermediary [3].

This work presents the Framework BlockProof that aims to help to guarantee the web content authenticity. The Framework features can be used by content providers to settle possible disputes inherent to differences between what a user says he has accessed and what the provider says he has published, since the provider can register what content is being published in a certain moment in time, it being possible to guarantee at any moment, if a piece of news, publication or post, in fact emerged from it.

Concepts of integrity, reliability and availability are fundamental in the analysis access evidence and content production. The main contribution of this work is to develop and validate a web application that has the purpose of being able to act, as this trusted third party is responsible for registering web content in such a way that it indisputably guarantees that the content published by a particular provider is in fact authentic. Content providers will be able to protect themselves from possible defamation raised against their reputation in addition to being able to offer their users a means to validate the history of updates performed on a given web page.

BlockProof also allows you to consult the entire records history that was made, which can be used by a user who wants to verify the news item origin, for example. In this way, it is possible to guarantee, at any time, whether a piece of news, publication or post has emerged from a certain content provider, which may propagate the information that it complies with the necessary rules that guarantee the possibility of attesting that, in fact, certain information passed through its servers. From the perspective of the content consumer, he will be able to search the entire update history of a given Web page, thus being able to prove, in court, if necessary, that certain information had been published on the Web.

Although we understand that the control of fake news is an important task (as proposed by BlockProof), up to this moment, it not based on our knowledge, as will be detailed in the related works of this article, any other work, or technology solution that proposes to solve this problem in a distributed manner. Our framework provides all the securities principles of any Blockchain based solution (authenticity, trustworthy, scalability and non-centralized operation) and also copes with a very important problem of fake news control compliance.

The rest of this article is organized as follows: Section 2 presents a brief theoretical foundation on the problem that underpinned the framework development. Section 3 presents some works related to the proposed problem resolution. Section 4 details the BlockProof proposal, and all the technologies and the agents functioning that are involved in the framework. Section 5 describes a tests Scenario that was considered in the framework validation, and Section 6 details all the test results. Finally, the conclusions and future works are described in Section 7.

## 2. Theoretical Foundation

Through Internet use and ubiquity becoming possible, a single human being, without leaving the place where he is using his computer, manages to be virtually at the same time in several places. Cyberspace becomes a fictional place, which we only have access to through the computer, and yet it is linked to reality by the use we have made of it, transforming it into an intermediary space between two realities [4]. The possibility that the Internet brings us opens loopholes for people with bad intentions to make use of this and commit malicious actions because they believe that anonymity is guaranteed. Cybercrime can be considered as a form of online diversion using technology, whether on a computer or smart phones. According to Sieber, a scholar of digital crimes, the history of “computer crime” goes back to the 1960s, when the first articles related to the topic became public [5]. These cases involved manipulation, sabotage, espionage and the illegal use of computer systems. In 1983, the OECD (Organization for Economic Cooperation and Development) defined that computer-related crimes are all illegal, unethical, or unauthorized behavior involving automatic processing and/or data transmission.

Digital crimes can be categorized into own digital crimes and improper digital crimes. Own digital crimes involve crimes in which the data are affected, such as: unauthorized access, illegal data acquisition and transfer, computer damage, viruses and their dissemination, disclosure or misuse of information, damage to the functioning of systems, social engineering and phishing. Improper digital crimes are committed with modern technology, are dishonorable, and they are crimes that are classified as: threat, fraud, participation in suicide, incitement and apology for crime, ideological falsehood, violation of rights copyright, trademark misuse and software piracy, child pornography and crimes without honor.

In the literature, we can find some research with the intention of helping in the fight against digital crimes, and they help to prove the integrity and authenticity of a document, image or video. According to [6], computer forensic expertise is the area responsible for investigating digital crimes, analyzing their facts and collecting data to use as evidence. This evidence needs to be collected in a way that does not compromise the obtained information. Forensic analysis is a multidisciplinary area in which, if we focus only on the obtention digital evidence of a cybercrime, the list of elements can be very long, which can include documents, emails, malicious software, messages and web pages, for instance. In others, all the evidence that was found can be used to prove some crime.

Disseminating fake content is not a complex task. There are several software programs that allow Web content to be downloaded and changed locally and capture content screens, changed locally, to then disseminate this image with distorted information associated with a Web publication over the Internet. The term fake news has become more famous in recent times, but this term has been used for hundreds of years. In the 19th century, English-speaking countries used this word to give title to big circulation rumors when fake news was disseminated in the media. Lately, producing and making fake news circulation feasible has become a market.

There are several crimes behind the fake news spread, such as illegal e-mail addresses, cell phone numbers and purchases, all with the spreading of fake content intention. A fake news disclosure can cause disastrous impacts, as mentioned in [7], when false information about a technology company sale was posted on a website. In that time, the fake news caused great volatility in company’s shares, generating an impact on the stock market.

In the Brazilian penal code, crimes of defamation (article 139), slander (article 138) and injury (article 140) are classified as crimes against honor. Such crimes can be made by hackers or even the spread of fake news and attributed to blogs, social networks, social networking sites, news agencies or e-mails, for example [8]—which can raise suspicions (and denigrate the reputation), questioning the suitability of people or companies.

Defamation crimes (article 139), slander (article 138) and injury (article 140) are classified as crimes against honor in the Brazilian penal code. Such crimes can be made by hackers or even the fake news spread and attributed to blogs, social networks, social networking sites, news agencies or e-mails [9]. These facts can raise suspicions (and denigrate the reputation), questioning the people or companies’ suitability.

Depending on the severity and the reachability that a forged content experiences, for a content provider to prove that he did not disclose that information, it may not be trivial. While the burden of proving the crime is legally incumbent on the prosecution, the destruction of reputation (and social cancellation, to quote a term that is currently in vogue) does not necessarily depend on a legal charge. Thus, how can a provider prove (in court or before society), indisputably that on that day/hour that news/information was not being made available on their servers? On the other hand, can a user, who is browsing the Internet, prove that he saw certain information, which may contain untrue content, of a personal nature, or some information that he would like to prove that was disclosed (such as, the value of a particular object on a shopping site) and that, after a failure on the site, it became different from what it was previously? Is it possible to offer, in a safe and indisputable way, a mechanism that allows for consulting the entire history of publication of Web content, including its updates? The search for a solution that responds positively to the questions raised above stimulated the studies of this work. To this end, we sought to use Blockchain technology, which has been growing in popularity recently, classified with the potential to modify the economy and business in the coming years.

Blockchain’s technology gained greater notoriety in 2009, after the launch of the whitepaper by Satoshi Nakamoto [10], in which the cryptocurrency Bitcoin was presented. Thus, Blockchain and its variations have been used to generate irrefutable computational evidence of the chronological order of transactions.

**Figure 1:**
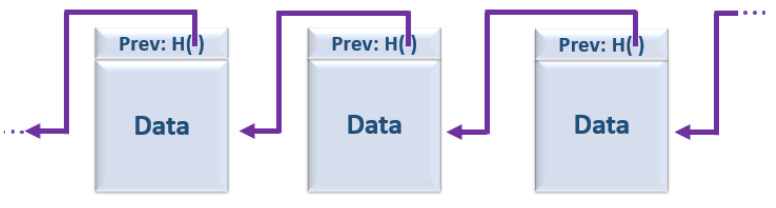
Blockchain data structure as a linked list of hash pointers.

The Blockchain term is used to refer to a data structure composed with an ordered block list. Its structure is basically formed by a linked list, using hash pointers, where this pointer gives the location of the information stored through a transaction. Each block in a Blockchain is “chained” back to the previous block, containing the previous one hash representation [11]. In Figure 1, you can see an illustrative representation of how the blocks that are Blockchain part are composed. If an attacker wanted to change the information of a transaction, he should change all the hash pointers of all successive blocks, which is computationally impossible, since the Blockchain can be made up of many blocks, and the storage is done in a decentralized way [12].

To be able to register a transaction in the Blockchain structure, the asymmetric cryptography is used with each member having a private key, which is used to sign their transactions, and a public key. This recording transaction method using asymmetric keys guaranteed a certain pseudo anonymization, since the identities of the participating parties are hidden from the network.

In the literature, it is customary to make an analogy between Blockchain and a distributed ledger, which is maintained in a consensual way and is validated by all parts of transactions on a network [13]. As mentioned above, transactions are grouped into blocks. In addition, the formation of blocks only occurs only if certain requirements are respected, and the most important for making a transaction effective is if it is verified as valid through some consensus mechanism. We can mention some better known consensus mechanisms, such as (i) proof-of-work, which consists of solving a computationally difficult challenge; and (ii) proof-of-authority, which is based on the validity of the transaction based on the reputation of Blockchain’s participating actors, among others. In addition, this validation step also provides that the records inserted in the Blockchain are considered safe and reliable since they are based on the hash calculation. Furthermore, it is the result of a transformation of the original information, known as a cryptographic hash, obtained through an algorithm and/or a mathematical function for a given input content, whose characteristic is that it is extremely difficult to infer the original information from the result of the algorithm/function. That is, a cryptographic hash is resistant to collision [14].

The creation facility and distributing content, much of it due to the technology advancement, a large amount of duplicated web content is find. According to studies carried out by Wang [15], approximately 80 percent of news on the web is not original, and they are partial or total existing content reproductions.

The search for a content similarity problem, also known as a nearest neighbor search, consists of finding an item that is the closest to a particular query item. It is a research topic on the rise as it can help identify various problems such as textual content plagiarism and copyright infringement detection.

In this work, we use the MinHash algorithm to achieve this purpose. MinHash is an LSH for similarity defined by binary vectors, and it is an approach capable of using constant storage independent of the length of the document as it makes use of the calculation of the hash of this content and produces an estimate of similarity. Similarity between two contents, such as Hamming, Levenshtein, etc., demands long computation times to complete the comparison between documents [16].

The difference with Minhash is that it seeks similarity between documents by calculating the sets intersection through a random sampling process. The main approach adopted by this method is to apply hash functions to each text pair in the documents, storing the value minimum for each of the functions hash applied. For the amount of content you want to compare to determine the overlap, it will take at least O (n + m) time to define the overlap between two documents [17].

Jaccard’s Similarity Coefficient is a statistical measure used to calculate the similarity between two sets. It is calculated as the ratio of the relative sizes of its union and its intersection. Following the consensus, to validate whether two documents are exact copies, it would be enough to compare the two contents character by character and, if any divergence is found, it could be concluded that they are not equal [18].

When referring to publications made mainly on the internet, the contents may not be identical but share large portions of their texts. As the goal in Minhash is to find the similarity index between documents, they may be considered similar if the similarity between them is greater than a given index.

## 3. Related Works

In the literature, there are several applications based on Blockchain, which involve voting systems [19], banking and financial [20,21], monitoring of food production and transport logistics [22,23], and many others. Most of them with the objective of making use of the benefits derived from technology as a guarantee of authenticity, immutability, transparency of registered information and so on. However, little is found to verify the authenticity and non-repudiation of records of Web content, as well as the history of all updates that have taken place in each piece of content.

The proliferation of misleading information disseminated in the media daily (such as social media feeds, news blogs, online newspapers) has become a challenge to identify reliable sources. Most of the approaches found for detecting false or manipulated content in some way, which are disseminated on the Internet, generally use crowd-sourcing techniques, as presented in the work of Pérez et al. [24] and the one proposed by [25], or protocols that prevent the spread of false news, as proposed by Sun et al. [26] and by Qayyum et al. [12]. In the latter, the authors describe, at a high level, without details or defining a system, how Blockchain technology can be used to detect and mitigate the spread of fake news. The others [24,25,26] depend on crowd participation to reach their objective. Besides that, it is worth to mentioning that none of them proposes to use Blockchain to increase the security and bring decentralization and scalability to the solution.

There is a platform called Politifact (https://www.politifact.com (accessed on 18 November 2021)) on which the journalists conduct several experiments extensively where some involve artificial intelligence and Deep learning techniques to determine the news accuracy. This platform has a consuming resources disadvantage with considerably high computations for processing a large data volume to determine the news accuracy, in addition to the concept used for classifying the news as: True, Mostly True, Half True, Mostly False, False, Pants on Fire, but this classification type can generate doubts.

The Original.my platform (https://originalmy.com (accessed on 18 November 2021)) is a system that offers an interface to a Blockchain platform, which allows future registration and verification of content. The platform allows you to register content, such as: statements, reports, images or any other type of document. There are several services associated with the Original.my platform for which, after sending the content to be registered, there is some generated information associated with the artifact such as a digital signature of the document, including the digital signature date and time registration on the Blockchain, date and time of registration, transaction code and the transaction digital certificate. On the platform, it is possible to check whether or not a file is on the Blockchain. The document is the key to the register.

In [27], Oliveira et al. present some platform services experiments that are offered, including the “bit Registry”, which proves the digital documents authenticity, and identified that, when making a small change in the input file, the hash to be generated will be completely different, as anyone would expect for a platform like that. The Original.my platform also offers the service “PacWeb ”, which acts in a similar way, but focused on web content. In this case, the “key” of the registration is performed from the “permalink” (Permalink is generally used by Web servers to customize the URL that points to a specific entry of the content. For example, a URL www.xyz.com/index.aspx?ref=0110 (accessed on 18 November 2021) can have the permanent link www.xyz.com/document/RG/0110/ (accessed on 18 November 2021)).

Blockchain technology is also used to guarantee the educational diploma’s authenticity since the fake diplomas’ numbers are a resource used very often by people who do not have qualifications but are looking for work. The costs to validate the diplomas’ authenticity are high, in addition to taking a long time for the institution to certify the authenticity of the document. The work exposed in UntungRahardja’s article explores technology for this purpose. However, the approach used by them implies that the students themselves publish their certificates on the blockchain where the company would consult this record when needed; this could be a problem if the student forgot to send the document to the blockchain. The work does not show in much detail how the proposed system would work at a more technical level [28].

Shantanu Sarode’s work addresses the manipulation of images and documents’ ease, citing some software that helps make these changes so that it is not possible to perceive these changes using the human eye. In his proposal, they use the neural network and blockchain concept to help verify the document’s authenticity by calculating the document’s hash. However, nothing was addressed about implementation details of the proposed web application [29].

The works that were found with proposals that are intended to guarantee the content authenticity present some worrying points when referring to web content. Most works encompass crowdsourcing techniques that require a lot of computational resources that are unfeasible to be implemented on a large scale or do not deal with the fact that the pages have dynamic or static content. Most of the approaches found to detect false or manipulated content in any way, which are disseminated on the internet, generally use crowdsourcing techniques, as presented in the work by Pérez et al.  [24]. In the experiments carried out by Pérez to guarantee the veracity of the news, they were manual checks carried out on the contents of the news that encompass tasks to cross-reference the source of the news. Once in possession of these results, a linear classifier was used, leading the assessments to increase the degree of validations in cross-references, and these data served as metrics for implementing learning algorithms. This approach is dependent on crossing references, and this crossing can not fit all existing news. The validation time of responses from returns from learning algorithms would require careful analysis to determine the accuracy of the algorithms used.

## 4. BlockProof Framework Proposal

The Ethereum platform was used, which uses a single canonical computer called the Ethereum Virtual Machine (EVM), where everyone on the Ethereum network agrees to maintain a state of that computer. Any participant can make a request to this canonical computer where they will perform the pertinent calculations and, once the other nodes of the network verify, validate and execute these calculations, a change in the EVM state is performed and propagated throughout the network. It uses a native cryptocurrency, Ether, which allows the existence of a market offering an economic incentive for participants to validate the requested transactions and to provide computational resources for the network [30].

The Framework enables content providers, responsible for hosting news portals, e-commerce, etc. to register the contents that are associated with a given URL at a given time. This feature provides greater reliability to users, content consumers from this provider, since it is possible to guarantee that the published information was originated by the provider that claims to have published it, thus protecting itself from content tampering attacks and possible Fake News dissemination.

Before going into the more technical details about the framework, it is important to keep in mind the difference between static and dynamic web pages, which will be mentioned in this article. A static website is generally smaller, informative web pages with no user interaction, commonly written only with HTML (HyperText Markup Languag) and CSS (Cascading Style Sheets), the final content of which is displayed in the user’s browser. A dynamic website, on the other hand, has more functionalities, interactions with the contents displayed on the page and can use combinations of other language scripts that are executed on a server before being displayed in the user’s browser. This feature makes it possible to create more complex systems, with more frequent updates.

Throughout the article, the term publisher will be used to refer to the person responsible for making the records in BlockProof, and the term consumer to refer to the person responsible for consulting a particular record in the application. The BlockProof contract, written in Solidity, consists of a struct that we call Transaction, and it contains all fields that will be stored in the Blockchain, along with a variable (urlHash) responsible for storing the URL hash, which will be informed by the publisher when making an application registration. This struct is associated with a data structure in Solidity known as Mapping, which is a structure that relates a key to a value. This data structure is associating to a string (urlHash) the entire struct with the data that will be sent to the Blockchain. We can say that the urlHash string will be the “primary key” for insertions and queries in the application. The contract has 13 functions, which will be explained in more detail during the description of the functioning of the prototype of the developed application. The solidity contract was “compiled” using the truffle.

**Figure 2:**
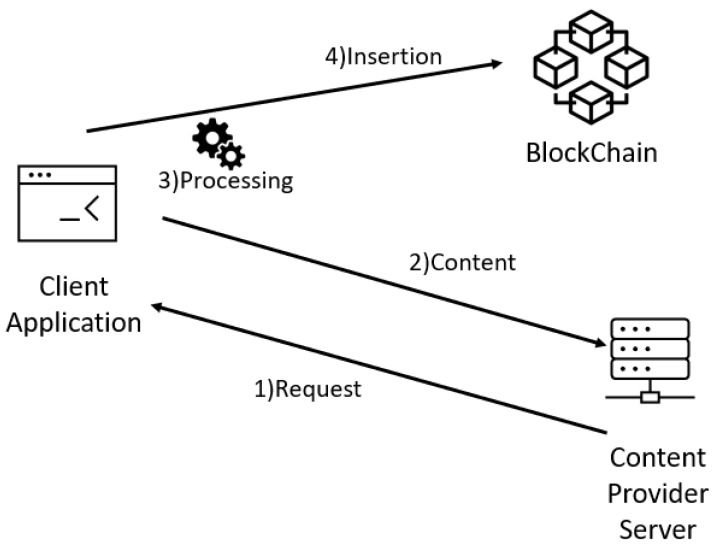
Insertion flow in block proof.

For a publisher to register with BlockProof, it is necessary to go through four main phases, as illustrated in Figure 2 and detailed below. As an example, let us say which publisher wants to insert the following URL in Blockchain: http://www.ic.uff.br/index.php/pt/pos-graduacao (accessed on 18 November 2021).

The **first phase (Request)** is the stage in which a user, responsible for the publishing server, authenticates with the tool (using existing user data and Ethereum account). After validating the data, the user is redirected to the registration screen, where he must enter the title and the URL that points to the content, and he wants to insert into the Blockchain. After standardizing the reported data, calls are made to contract functions that (i) calculate the received URL hash (sha256) and (ii) use a Python *request* library to retrieve all code from the page content that is related to the informed URL, the return being stored in an object, and then moving on to phase 2.

**Figure 3:**
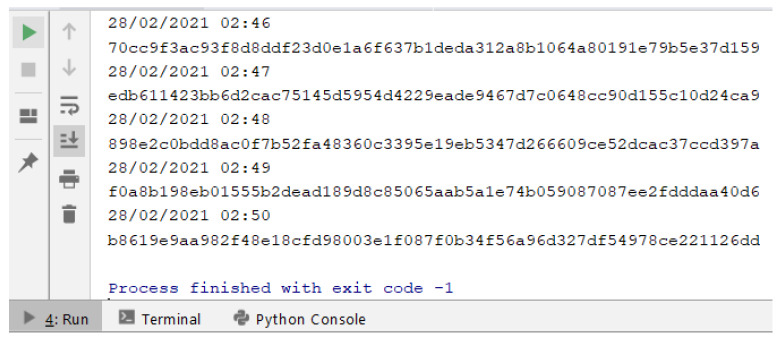
Hash values of content associated with different requests for a “dynamic” URL.

**Figure 4:**
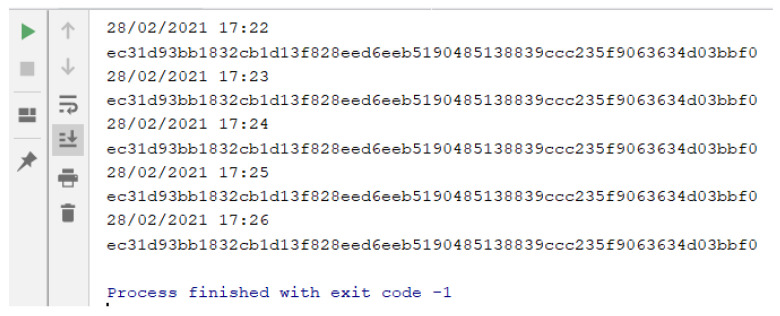
Hash values of content associated with different requests for a “static” URL.

In the **second phase (Content)**, the content of the retrieved Web page (stored in the object at the end of phase 1) also submitted to the hash calculation (sha256). That way, we have the hash of the URL to be inserted and the hash of the content returned by the server for that given URL. These data are not enough, due to the possibility of the pages being dynamic. Note that the hash results for content retrieved in different requests, for the same URL, can be different, even though they are visibly identical. This is since the pages may show differences (substantial or minor) for each request, such as: some type of timer, advertising images being varied with each request, customization from cookies, among other possible details. For example, a hash (sha256) of the content of two urls has been calculated every minute. The content associated with the first URL (http://www.ic.uff.br/index.php/pt/pos-graduacao (accessed on 18 November 2021)) can be classified as dynamic, while the content of the second URL (https://docs.python.org/3/library/time.html (accessed on 18 November 2021)) is static. The results of executing these commands can be seen Figure 3 and Figure 4.

**Figure 5:**
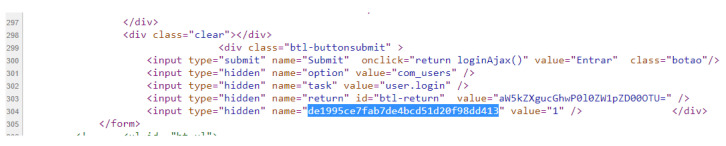
Source code of the page that results in the different hash values.

In Figure 3, we can see that the content hash value is different for each request, which is inferred, by the very definition of the hash calculation, that there was a variation in the input object, which resulted in a distinct hash value. In Figure 4, what can be seen is that the same hash value is the result of several requests for the content of the same URL. Analyzing what changes took place in the “dynamic” page, the content of which is illustrated in Figure 5, the only difference is in a parameter of a form used on the page (highlighted in the image), which is associated with the session cookie. The fact that this source code is different with each request does not change the content that is being displayed to the end user.

In order to allow the Blockchain records to distinguish a significant change in the occurrence web page as described above, the similarity concept in the contents of the records was added to BlockProof. For this, the use of algorithms for this purpose was added to the framework, in this case Minhash. In [31], Christiani et al. describes the mathematical evidence to support the Minhash use to calculate the similarity content degree.
**Algorithm 1** Inserting a new record**Require:** title, url, autor
 1:$first_{h}ash_{U}RL_{C}ontent\leftarrow 0$ 2:$second_{h}ash_{U}RL_{C}ontent\leftarrow 0$ 3:minHashList1←0 4:minHashList2←0 5:**if**$first_{h}ash_{U}RL_{C}ontent=second_{h}ash_{U}RL_{C}ontent$**then** 6:   {insert record in blockchain} 7:**else** 8:   minHashList1←Lm1 {calculate minhash list for hC1} 9:   minHashList2←Lm2 {calculate minhash list for hC2} 10:   {calculate Jaccard index} 11:   {insert records in blockchain} 12:**end if**


We can see in the Algorithm 1 the pseudo algorithm of the blockchain insert function. BlockProof’s **third phase (Processing)** foresees that, for each URL record, two retrieval content will be performed in sequence, and the hashes (sha256) of the received content will be computed. If the results of these hashes are different, further processing to calculate the similarity is performed, comparing the two versions retrieved from the contents by calculating Minhash [31] between them. Two lists are created, one list for each version of the content, which will store the values resulting from the following procedure:

- **Step 0—Generate fixed-size subsets of the content.** In this case, we use subsets of 30 characters. Therefore, they are initially generated *N* subsets of each recovered content, the number of subsets of the content being *i* given by $N_{i}=\frac{T_{i}}{30}$, where $T_{i}$ represents the size of the content *i*, with i={1,2};
- **Step 1—Transform each subset using a hash function.** Each one of *N* subsets are transformed into new subsets, based on the results of the hash function calculation (in this case, MD5 was used);
- **Step 2—Store the lowest value of the function applied to each set.** After applying the hash calculation (MD5) to each subset, the lowest of all *N* MD5 computer in a list of minimum values;
- **Step 3—Repeat the steps above many times.** The steps described above are repeated 100 times. The transformed subset (containing the results of the transformation from the hash in the current interaction) is again submitted to “Step 1”, until 100 minimum hash values are obtained.

The pseudo code of the MinHash calculation can be found in the Algorithm 2:
**Algorithm 2** calculate minhash**Require:** req
 1:$list_{m}d5\leftarrow 0$ 2:$list_{t}ok\leftarrow get_{f}eatures\left(req,30\right)$ 3:$list_{h}ash_{m}in\leftarrow 0$ 4:rep←0 5:**while** rep< 100 **do** 6:   $list_{m}d5\leftarrow hash_{m}d5\left(list_{t}ok\right)$ 7:   $list_{h}ash_{m}in\leftarrow min\left[list_{m}d5\right]$ 8:   $list_{t}ok\leftarrow list_{m}d5$ 9:   rep←rep+1 10:**end while** 11:**return**$list_{h}ash_{m}in$


The two lists resulting from the algorithm described above consist of 100 minimum hash values, for each of the two distinct contents retrieved. Like Minhash [16], the similarity coefficient between these lists is then calculated, resulting in the content similarity coefficient. This coefficient is obtained by calculating the Jaccard similarity index, which divides the size of the intersection between the two lists by the size of their union. That is, consider $L_{1}$ and $L_{2}$, respectively, as being the two sets formed from the minimum hash elements of the lists of each of the contents. Jaccard’s similarity indicator, *J* is obtained as follows:

(1) $$J=\frac{|L_{1}\cap L_{2}|}{|L_{1}\cup L_{2}|}$$

Finally, in the **fourth phase (Insertion),** the data are recorded in Blockchain. BlockProof stores the following data: the hash 256 of the url, the hash 256 of the content of the url, if it is a dynamic page, the second value obtained for the second time of the content associated with the url, the publisher’s identification, the day and the time of insertion, the news title, all two minimum MD5 hash lists obtained as described above, and the Jaccard index. This registry is now offered for future queries for any user interested in the history of records (updates) associated with a given URL.

**Figure 6:**
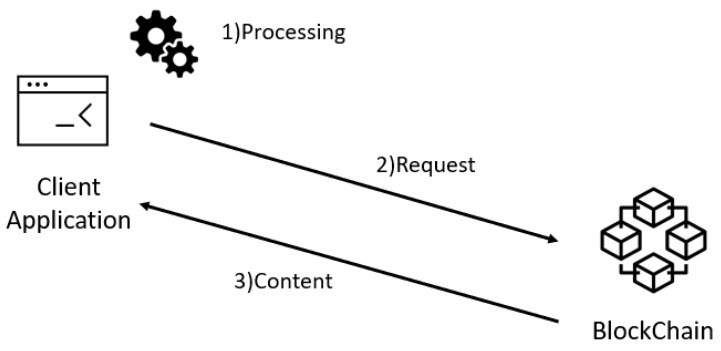
Query flow in BlockProof.

When the user wants to query the history of a record associated with a certain URL, he/she need to **(1) Process** the URL computing its hash 256 and send **(2) Request** to the BlockProof. The query is processed by the Blockchain and a response will be returned to the querier. The **(3) Content** resulting from this query basically consists of the entire record history indexed by the hash of that URL ever included in the BlockProof. The query flow is summarized in Figure 6.

## 5. Experiment Scenarios

**Figure 7:**
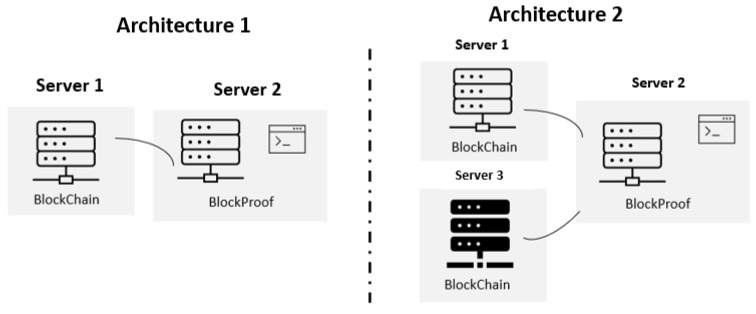
Test environment architecture.

The tests were performed with two Architectures that can be seen in Figure 7. Architecture 1 is composed of two servers where one hosted the application and the files responsible for the tests that will be described later, and the other hosted the Blockchain technology. Architecture 2 is composed of three servers where one hosted the application and the files responsible for the tests and the other two each hosted a Blockchain structure technology. All servers have operating system Ubuntu, with Linux version 5.4.0-77-generic, gcc version 9.3.0, ubuntu 20.04 and were in the same VLAN (Virtual Local Area Network)

The used test performs tool measures applications performance using the concepts of requests, which are basically messages exchanged during a client–server communication flow. Sending messages is initiated by the client responsible for originating the communication, commonly represented by web browsers, which uses the HTTP (Hypertext Transfer Protocol), which is a basic protocol for obtaining resources between any data exchanges operated over the internet. The protocol has several methods that tell the server what type of action the client wants to take. Request messages are answered by the servers through replies to messages as replies.

Inside, the script file contains all the information to carry out the insertion tests and queries in the Blockchain through the BlockProof application. The methods and technical terms necessary for the framework construction which were informed, which would be the methods of the HTTP protocol used and which data would be sent to the application, perform the simulations. One thousand URLs were chosen in a random way, encompassing static and dynamic URLs to run the tests. The users’ simultaneous numbers that will be used for the test were informed, and the user number that will be added to the test per second until reaching the specified number in the previous option.

The test tool simulates a user interaction with functions that were specified in the test file. This is useful to measure the application performance, and we can say that the number of clients is equivalent to the number of calls to these functions, in the BlockProof application, responsible for performing the Blockchain Insertion/Consulting. In the way the test was formulated, the user number is equivalent to the number of functions BlockProof application calls, which is equivalent to the request number that the client (browser) will make to the BlockProof application during the tests. Thus, we will call the simultaneous user numbers that will be used for the test, as the limit simultaneous requests number (LRS) that you want to simulate, as well as the user numbers that will be added to the test per second until reaching the number specified in the previous option as a request increment that will be performed every second (IRS).

At each execution batch, we will have as total load the value N, which will be incremented every second with the request value to be executed simultaneously until reaching the defined LRS value, as explained above. The total load value will be N × 1000 (value of URLs specified in the test file, which, for this experiment, 1000 URLs were used, classified as static and dynamic). Keeping in mind the insertion function, the first batch will be composed of 1000 URL insertions and the next ones will be updates of those 1000 URLs as explained above. Having defined LRS, IRS and total load, we have the following test scenarios that were performed in this work for both defined Architectures:

- Scenario 1: LRS = 10 and IRS = 1, 1000 new simultaneous insertions, in the first second, and an increment of 1000 simultaneous updates per second, until reaching the total load of 10 × 1000 simultaneous insertions.
- Scenario 2: LRS = 100 and IRS = 10 that is 1000 new inserts + (9 × 1000 updates) simultaneously, in the first second, and an increment of 10,000 simultaneous updates per second, until reaching the total load of 100 × 1000 simultaneous insertions.
- Scenario 3: LRS = 1000 and IRS = 100 that is 1000 new insertions + (999 × 1000 updates) simultaneous, in the first second, and an increment of 100,000 simultaneous up-dates per second, until reaching the total load of 1000 × 1000 simultaneous insertions.
- Scenario 4: RS = 10,000 and IRS = 1000 that is 1000 new insertions + (9999 × 1000 updates) simultaneously, in the first second, and an increment of 1,000,000 simultaneous updates per second, until reaching a total load of 10,000 × 1000 simultaneous insertions.

## 6. Experiment Results

This section presents all the results obtained from the tests performed at the scenarios described above. The following graphs will provide an application’s behavior pictorial view throughout the test’s execution. We use line graphs, the *x*- and *y*-axes represent the batch current values and the average response time, in milliseconds, which indicates the average request return time that was sent to the application to be effective for the corresponding batch in the BlockProof application. The graphs also show the curve related to the 95% percentile, which are measures that divide a sample of values, ordered in ascending order into one hundred parts, often used to measure the degree of something acceptance. For example, let us assume that 80% of users need to have a 5 s response time. If the 80% percentile, after sorting the data obtained during the tests, is 7 s, it means that 80% of users obtained a time greater than the stipulated.

**Figure 8:**
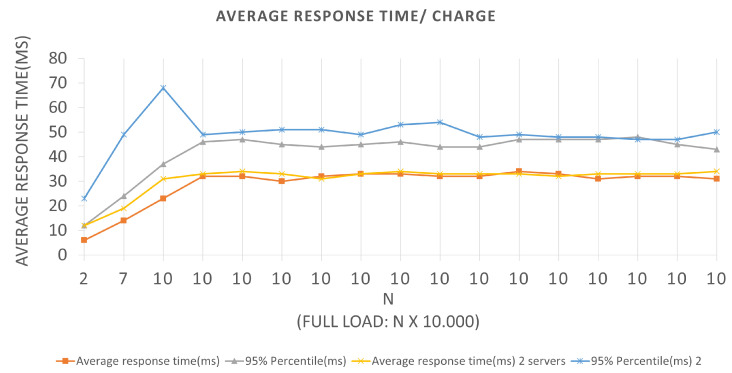
Results of the experiments for insertion loads.

The tests related to Scenario 1, the graph represented in Figure 8 has an LRS = 10 and an IRS = 1. They were performed for both Architectures, and we can extract the following data by looking at the graph: In Architecture 1, for the first batch plotted on the graph, we have an N value of 10, a total load of 10,000, an average response time of 23 ms, and a 95% percentile of 37 ms. In other words, for BlockProof performing 1000 new insertions and 10,000 updates of these same URLs, the application takes an average response time of 23 ms for the total load to go through the entire algorithm of the insertion function and return for each simulated “user” the response of this record.

As a tool used for testing, it preserves cookies between requests, thus making it possible to perform logins and consume remote methods that depend on an active user session, for Architecture 1, if the BlockProof server tries to meet the total load number for this first batch (10 × 1000 users (requests)), 95% of users (requests) will have their registration request answered in 37 ms. For the same batch in Architecture 2, the application presented an average response time of 12 ms and a 95% percentile of 23 ms. In other words, for the BlockProof application to perform 1000 new insertions and 1000 updates of these same URLs, it takes an average response time of 12 ms for the total load to go through the entire insertion function algorithm and return to each simulated “user” the response of this record. If the BlockProof server tries to meet the total load number for this first batch (2 × 1000 users (requests)), 95% of the users (requests) will have their registration request’s response satisfied in 23 ms.

**Figure 9:**
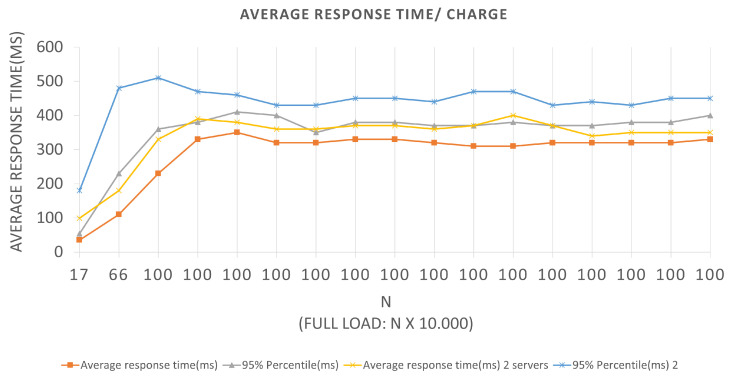
Results of the experiments for query loads.

In the time interval in which during the test, 0 failures per second (FS) were reported on average, that is, all requests made were supported by the application, and an average total of 294.9 requests per second (RPS) were bagged, for the first Architectures, and an average total of 283.8 requests per second for the second. With a total load of 10,000 (ten thousand) simultaneous requests per second, an application presents a relatively good performance, since it does not present considerable failures. The Scenario 2 tests graph represented in Figure 9 has an LRS = 100 and an IRS = 10. An increment of 10 × 1000 requests per second was performed until reaching 100 × 1000 requests per second. They were also performed for both Architectures, and we can extract the following data by looking at the graph.

In the first batch for Architecture 1, we have a value of equal to 100, a total load equal to 100,000, an average response time of 230 ms, and a 95% percentile of 360 ms. In other words, for the BlockProof application to perform 1000 new inserts and 99 × 1000 updates of these same URLs, it takes an average response time of 230 ms for the total load to go through the entire insert function algorithm and return for each simulated “user” the response of that record. For Architecture 1, if the BlockProof server tries to meet the total load number for this first batch (100 × 1000 users (requests)), 95% of the users (requests) will have their registration request’s response satisfied in 360 ms. A 100 batch for Architecture 2, the application presented an average response time of 330 ms and 95% percentile of 510 ms. In other words, for the BlockProof application to perform 1000 new insertions and 100 × 1000 updates of these same URLs, it takes an average response time of 330 ms for the total load to go through the entire insertion function algorithm and return for each simulated “user” the response of that record.

**Figure 10:**
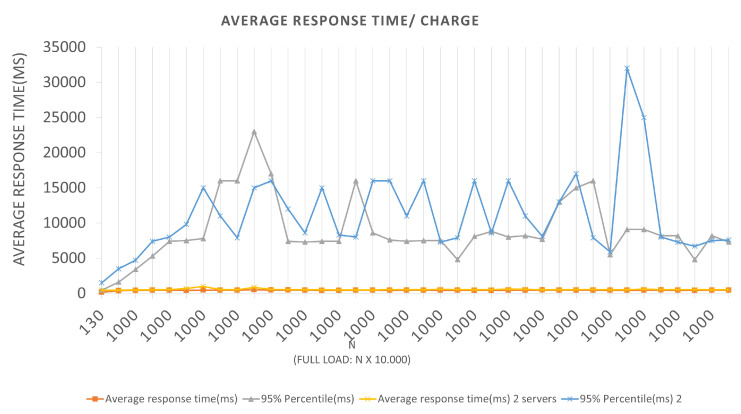
Results of the experiments for query loads.

**Figure 11:**
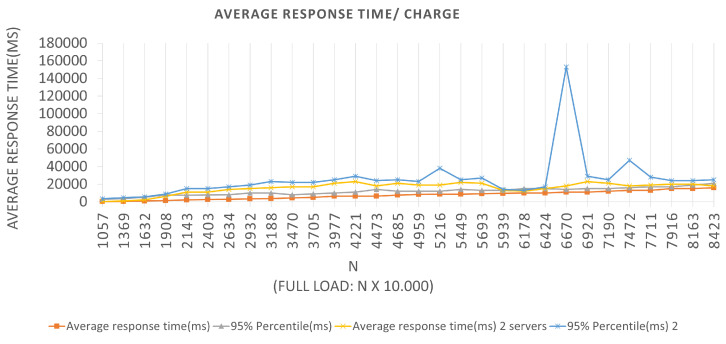
Results of the experiments for query loads.

For Architecture 2, if the BlockProof server tries to meet the total load number for this first batch (100 × 1000 users (requests)), 95% of the users (requests) will have their registration request’s response satisfied in 510 ms. In the interval time in which the test was conducted, 0 failures average per second (FS) were reported in the two Architectures, that is, all requests made were supported by the application, and a 294.9 total average requests per second (RPS) were completed, for the first Architecture, and an 271.7 average total requests per second. It also performed relatively well, in the configurations, a servers view was used, and the failure numbers were not considered. For Scenarios 3 and 4, Figure 10 and Figure 11, respectively, an LRS of 1000 and an IRS of 100 were stipulated for Scenario 3, and an LRS of 10,000 and an IRS of 1000 for Scenario 4. The analysis of the plotted points in the graphs follows the same logic described above for the two previous scenarios.

We can see in the graphs that the tests in Scenario 3 presented a higher 95% percentile value for both Architectures. Several tests were carried out for this Scenario, and this same anomaly was identified when a constant batch rate was reached. One hypothesis to justify this behavior, since the errors reported were Connection timed out due to the time of waiting for the response to the request to have been high for the capacities of the servers used in the tests. This fact generated a longer response time, leading the server to reject new requests because it did not finish executing the previous ones. Queries records Tests to the Blockchain, through the Block-Proof application, following similar Scenarios adopted for the previously mentioned insertions. Remembering that, to perform a particular query in the Blockchain, two contract functions are activated—one to query the number of records linked to the URL hash being queried—the number of queries required to return all parameters that were recorded for each value to iterate over until the limit is reached.

**Figure 12:**
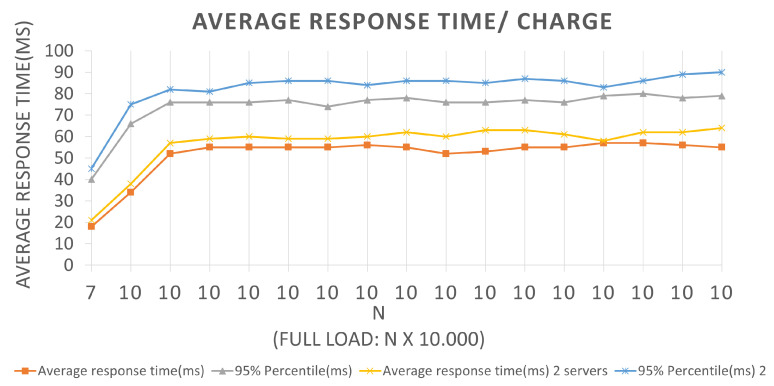
Results of the experiments for query loads.

Figure 12 represents the curves for Scenario 1 tests. The *x* and *y*-axes represent the current batch values and the average response time, in milliseconds, which indicates the average return time of the response to the query message sent to the application to be effective for the corresponding batch in the BlockProof application.

The graphics interpretation is like the interpretation performed for the graphics corresponding to the insertion requests. Choosing the first plotted point for an example, in Architecture 1, where only test parts that were performed used two servers, for a batch equal to 10, a 34 ms average response time was obtained with a 66 ms 95% percentile, and for test Architecture 2, with three servers, the application reported an average response time of 38 ms and a 95 % 75 ms percent. Considering that the entire history of records associated with the URL hash was returned, the application presented an almost constant behavior when it reached the total load of ten thousand requests per second and presented 0 failures during its execution.

**Figure 13:**
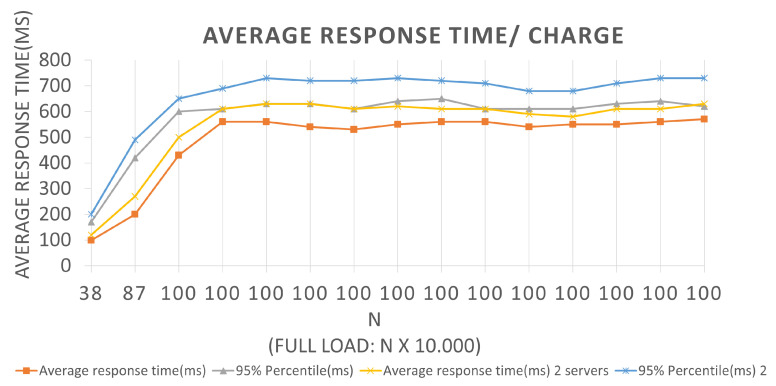
Results of the experiments for query loads.

In the tests related to Scenario 2, the graph represented in Figure 13 has an LRS = 100 and an IRS = 10. An increment of 10 × 1000 query requests per second was performed until reaching 100 × 1000 requests. Tests were performed for the two Architectures, and we can extract the following data by looking at the graph: In Architecture 1, for the first batch, we have a value of N equal to 100, a total load equal to 100,000, i.e., 100,000 query requests will be performed at once from simultaneously. For this full load value, an average response time of 430 ms and a 95% percentile of 600 ms were reported. That is, for the BlockProof application to perform 100,000 query requests, to query the entire history of existing records in the Blockchain for the URLs contained in the test file, it took an average response time of 430 ms for each request to be met where 95% of users (requests) obtained the response of their registration request answered in 600 ms.

**Figure 14:**
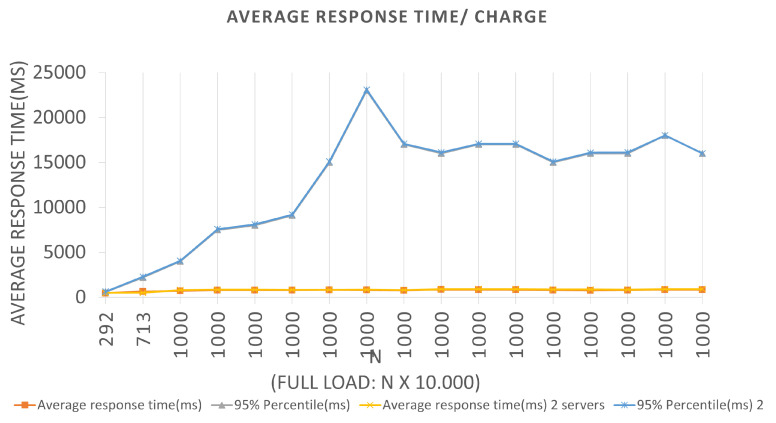
Results of the experiments for query loads.

**Figure 15:**
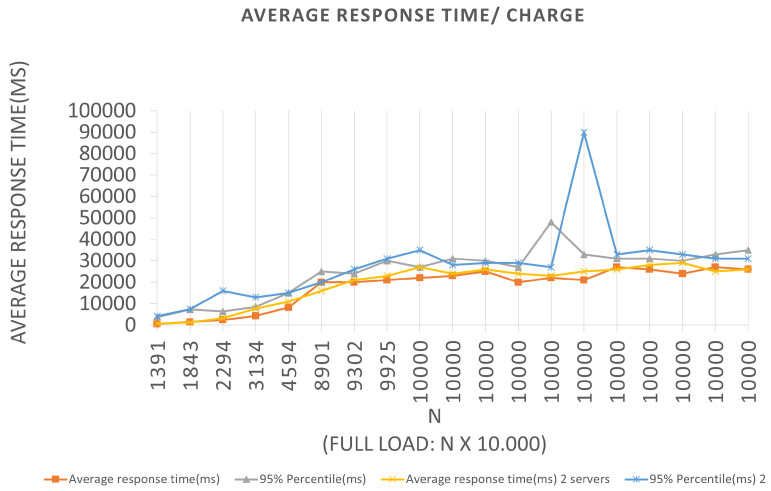
Results of the experiments for query loads.

Similar interpretations can be performed for the other plotted points and for the graphs corresponding to Scenarios 3 and 4, which can be seen in Figure 14 and Figure 15, respectively. The graphs show the application behavior for an LRS = 1000 and an IRS = 100, and for an LRS = 10,000 and an IRS = 1000, in that order. We can observe for Scenario 3 that the same “anomaly” was identified and a justification hypothesis like the one used for the insertions can be adopted since the reported error was similar. The errors reported were from Connection timed out because the wait time for the response to the query request message has been increased for the capacities of the servers used in the tests. This fact may have generated a longer response time, causing the server to reject new requests since it did not finish executing the previous requests.

## 7. Conclusions and Future Work

This work exposed a latent problem which is the lack of efficient approaches to irrefutably guarantee the integrity and the authenticity of web content. Despite the absence of solutions, it is a crucial task to help in the digital crime fight in order to prove the trustiness of a published document, image, or video. Among all the digital crimes, fake news certainly is one the most recurrent ones and needs to be mitigated.

In this paper, the BlockProof was presented, a framework for verifying web content authenticity and integrity that offers a solution for content providers to register Web content, regardless of whether the page has dynamic or static content, in addition to enabling the consultation of the history of all records made for a given URL. Such kind of solution has become important nowadays as a way to cover the gap in the fight against fake news, for example. We understand that such kind of solution may be useful to data producers/providers to provide evidence that they are in compliance with fighting against fake news, for instance.

The aim of the prototype that was developed is that the application can be used for any web pages with static and dynamic content. This application can provide an authentic and reliable evidence of what was actually published, since the content has been inserted into the Blockchain, ensuring that the information was actually published by the content provider. A contract was drawn up that was responsible for interacting with Blockchain, capable of identifying whether, for a given URL hash, there is already a record contained in the Blockchain, with the appropriate meta data related to the web page that make it possible to identify the person responsible for having registered the content. The application allows for obtaining, when necessary, the entire history of records linked to the stored URL.

There are several Blockchain-based applications in order to make use of the benefits derived from technology, but little is found to verify the authenticity of Web content records as well as the history of all updates that have taken place in each Web content. Thus, in this sense, BlockProof is a novel and a significant contribution. The prototype implementation was evaluated throughout several experiments in different scenarios. Obtained results show that a satisfactory scalability can be achieved even with a limited infrastructure.

As future work, we foresee that the solution may be evaluated against different Blockchain technologies. It could increase even more the system’s scalability and/or bring more flexibility for options to the users.

## Abbreviations

The following abbreviations are used in this manuscript:

IP	Internet Protocol
URL	Uniform Resource Locator
DNS	Domain Name System
OECD	Organization for Economic Cooperation and Development
EVM	Ethereum Virtual Machine
HTML	HyperText Markup Language
CSS	Cascading Style Sheets
VLAN	Virtual Local Area Network
HTTP	Hypertext Transfer Protocol
LRS	Limit Simultaneous Requests
IRS	Increment Requests per Second
RPS	Requests per Second
FS	Failures per Second
